# Facilitators and Barriers to Successful Collaboration in Guideline Development: Experiential Insights of Collaborative Guideline Developers

**DOI:** 10.1002/gin2.70036

**Published:** 2025-08-26

**Authors:** Chatura Prematunge, Amanda Graham, Christina Ventura, Robyn Temple‐Smolkin, Emma McFarlane, Aggie Bak, Pamela Ginex, Yasser Sami Amer, Michael Yi, Umer Nadir, Emily Senerth, Priya Jakhmola, Heba Hussein, Bright Huo, Rachel Christensen, Holger Schunemann, Murad Alam, Rebecca L. Morgan, Toju Ogunremi, on behalf of the Guidelines International Network Guidelines Collaboration Working Group

**Affiliations:** ^1^ Public Health Agency of Canada Ottawa Ontario Canada; ^2^ Guidelines International Network Pitlochry UK; ^3^ College of American Pathologists Northfield Illinois USA; ^4^ Association for Molecular Pathology Rockville Maryland USA; ^5^ National Institute for Health and Care Excellence London UK; ^6^ Healthcare Infection Society London UK; ^7^ Stony Brook University Stony Brook New York USA; ^8^ King Saud University Riyadh Saudi Arabia; ^9^ Northwestern University Evanston Illinois USA; ^10^ Society for Cardiovascular Angiography and Interventions Washington District of Columbia USA; ^11^ Centers for Disease Control and Prevention Atlanta Georgia USA; ^12^ Department of Oral Medicine and Periodontology, Faculty of Dentistry, Cairo University Cairo Egypt; ^13^ Dalhousie University Halifax Nova Scotia Canada; ^14^ McMaster University Hamilton Ontario Canada; ^15^ Case Western Reserve University Cleveland Ohio USA

**Keywords:** barriers, clinical practice guideline, collaboration, facilitators, guidance, guideline development, guidelines, policy, thematic analysis

## Abstract

**Objective:**

The current study aimed to elucidate themes that contributed to successful and unsuccessful collaborative guideline development between organisations.

**Study Design and Setting:**

The reporting of qualitative methods was checked against the Consolidated criteria for reporting qualitative research (COREQ) checklist. A purposive sampling approach was used to recruit participants with experience or affiliations in collaborative guideline development. Participants completed a pre‐interview questionnaire and took part in semi‐structured interviews. Interview transcripts were coded using inductive thematic analysis methodology.

**Results:**

The thematic analysis of guideline developer interviews (*n* = 14) identified three interconnected theme sets and secondary sub‐themes. Misalignment of collaboration purpose and scope, misalignment of guideline development methodologies, and perception that such collaborations require more time and effort were identified as barriers. Technologies and templates that support collaborative work, initial scoping and planning among collaborators, organisational guideline topic prioritisation work, culture of collaboration, and recognising collaboration benefits were identified as facilitators. Cross‐cutting themes, which appeared to either enhance or hinder collaboration based on interviewee context, included past collaborative experiences, publication of completed work, and levels of knowledge and training among collaborators.

**Conclusion:**

This analysis demonstrated facilitators and barriers to collaborative guideline development are interconnected. Future research into collaborative guideline development should look towards validating these findings in a more geographically diverse sample and investigate the mechanisms through which barrier, facilitator and cross‐cutting themes contribute to guideline collaboration between organisations. Further understanding of factors that can impede or expedite successful collaboration provide opportunities to identify effective strategies for overcoming perceived barriers to collaboration. Findings from this study can be used to bolster collaborative efforts by encouraging alignment in processes, methodology, and expectations, as well as promoting collective knowledge and resource sharing between collaborators within different guideline development organisations.

## Summary

What is new?

- Barriers and facilitators to collaborative guideline development are interconnected.
- Alignment in methodology, processes, and expectations is critical for success.
- Collaboration is fostered by supportive technology, planning and sharing of resources across guideline development organisations.
- Successful collaborative processes can bolster efficient use of resources and improve guideline dissemination and use.

## 1 Introduction

Clinical practice and public health guidelines (collectively referred as guidelines hereafter) present systematically developed statements and recommendations to support evidence‐based health decision‐making among practitioners and patients or persons benefiting from the guideline [1, 2]. Guidelines aim to improve health outcomes, healthcare practices, and optimise resources [1, 2, 3, 4, 5, 6, 7, 8, 9, 10]. The production of quality guidelines are often coordinated by national or international organisations and professional societies, and requires participation and collaboration among different experts, user audiences, and impacted patients [10, 11, 12, 13]. In this paper we refer to collaborative guideline development to denote two or more organisations or professional societies working together to develop a full guideline, or guideline components.

The Guidelines International Network (GIN) Guidelines Collaboration Working Group (GCWG) is an international group of guideline developers who work together to develop tools for successful collaborations and build a community for sharing best practices and lessons learned [13, 14, 15]. In 2019, GCWG and the US GRADE Network conducted a needs assessment survey that identified perceived needs and challenges related to collaboration efforts among guideline developers [16]. The survey identified that difficulties reconciling differences in guideline methodology and the time required to establish collaborative agreements most significantly impeded collaboration between organisations. To further understand the implications of these challenges and gather real‐world examples, subsequent exploratory research was needed. The current study aimed to elucidate factors that influenced successful and unsuccessful collaborative guideline development through follow‐up interviews with the survey participants.

## 2 Methods

Reporting of this thematic analysis was checked against the Consolidated criteria for reporting qualitative research (COREQ) checklist to ensure comprehensive and transparent reporting [17].

### 2.1 Development of Pre‐Interview Questionnaire and Interview Guide

GCWG developed the pre‐interview demographic questionnaire and semi‐structured interview guide by drawing on the needs assessment findings, available literature and their collective expertise in collaborative guideline development (Appendix). Participants were requested to complete a standardised pre‐interview questionnaire independently a few days before engaging in the interview. The pre‐interview questionnaire collected participant demographic information, experience and affiliations in guideline development.

An interview guide directed all interviews and interviewer questions. A preliminary version of the interview guide was piloted using four mock interviews. Mock interviews informed interview guide refinement but responses were excluded from the analysis. The interview guide included an introductory script for the interviewer, semi‐structured questions and interviewer prompts to facilitate complete interviewee responses. The interview questions focused on individual experiences and insights on collaborative guideline development among organisations, staff expertise, availability of resources, experiences during the COVID‐19 pandemic, processes for managing conflict of interest (COI), peer review, publication, dissemination, implementation, updating or retiring guidelines.

### 2.2 Participant Selection

A purposive sampling approach was used to recruit participants with experience or affiliations in collaborative guideline development. Invitations to participate in interviews were emailed to individuals and organisations who participated in the needs assessment and therefore involved in guideline development. Only needs assessment participants who had consented to being contacted for follow‐up study participation were contacted to participate in interviews. If unable to participate, contacted individuals were invited to forward the invitation to a colleague at their organisation suited to discuss the topic. Up to three follow‐up reminders were sent between December 2022 and March 2023 to maximise participation. No individuals who agreed to participate in the interviews dropped out.

### 2.3 Semi‐Structured Interviews

Interviews were conducted by GCWG members within this study team acting as interviewer(s) and scribe(s) as detailed by Newcomer et al. [18]. Participants were informed their interviews would be recorded and transcribed, to which all participants provided consent. Interviews were conducted through pre‐scheduled virtual meetings facilitated by GCWG members on the study team.

No one other than the interview participants were present during each interview, which took on average 45 min to complete. No repeat interviews were conducted. Interview recordings were transcribed verbatim by a professional transcription service. All interview recordings, notes, transcriptions, and participant information were considered confidential study materials and stored within an institutional secure drive.

### 2.4 Qualitative Data Analysis

**Figure 1:**
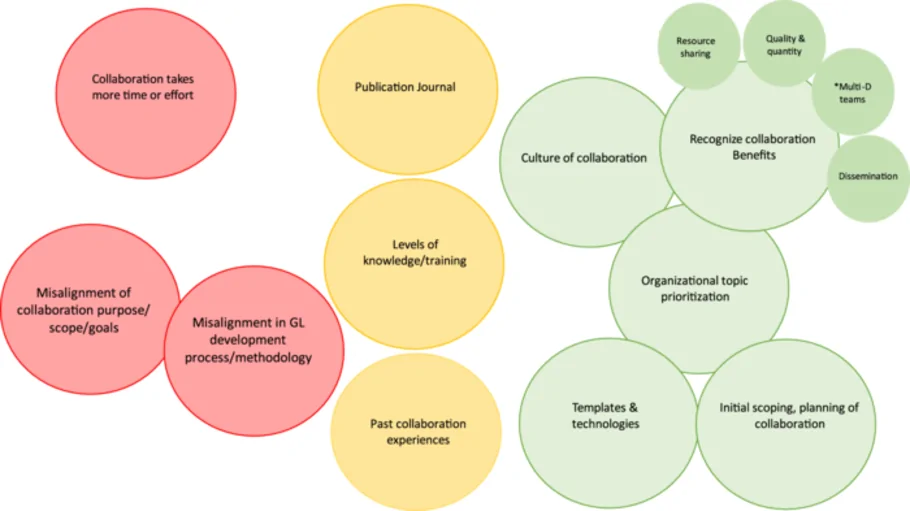
Themes map of facilitators and barriers to successful collaborations in guideline development: The thematic map was developed iteratively, considering critical elements of data visualisation to convey the key patterns observed in the data. Each set of themes were grouped to a common area on the map, and representative colours were assigned to each set of themes: barrier (red), facilitator (green), and cross‐cutting (yellow). Core themes were assigned a larger circle, while important associated sub‐themes were assigned smaller circles, latched to their parent theme on the map.

The aim of this qualitative analysis was to explore the insights and experiences of guideline developers to better understand collaboration in guideline development. De‐identified interview transcripts were analysed line‐by‐line by four coders (AB, AG, BH, CP) using inductive thematic analysis methodology described by Kiger and Varpio [19]. Coders independently familiarised themselves with the interview data, generated initial codes, and searched for emergent sub‐themes and themes using MS Excel spreadsheets. Following independent coding, coders collectively refined and organised the sub‐themes, defined and named broader themes through discussions until no additional unique themes or sub‐themes could be identified. Input from participants on the identified themes was not collected. Identified themes were graphically mapped to create a thematic map of barriers, facilitators, and cross‐cutting themes (Figure 1).

### 2.5 Ethics Considerations

The interview project was reviewed and waived by the McMaster University Research Ethics Board (Hamilton, ON) as a quality improvement activity which addresses tool development designed to assist the specified organisations in collaborating.

### 2.6 Author Reflexivity

The study objectives aligned with GCWG interests and were shared with participants before starting the interviews. All interviews and data analysis were conducted by the GCWG study team, who are GIN members, experienced and interested in collaboration during guideline development.

The guideline development community is small and well‐networked, with increasing number of guideline development organisations becoming GIN members. As a result, a few interviewers and interviewees had a degree of professional familiarity with one another. Interviewers adhered to a pre‐established interview guide and script to minimise bias and enhance transparency. The GCWG study team are experienced in collaborative guideline development and champions of such collaborations, therefore it is anticipated their opinions, insights and lived experiences are reflected in this thematic analysis.

## 3 Results

From the 24 invited participants, 16 guideline developers affiliated with 12 different guideline development organisations provided interviews (67% participation rate) between January and April 2023; 14 interviewees completed the pre‐interview questionnaires. Characteristics of interview participants who completed the pre‐interview questionnaires are provided in Table 1.

**Table 1:** Characteristics of interview participants who completed pre‐interview questionnaire.

Demographic variable	Number of participants, *n* (%)
Participates in guideline collaboration in current role (*n* = 14)	
Yes	11 (79%)
No	3 (21%)
Geographic region (*n* = 14)	Number of participants, *n* (%)
North America	10 (71%)
Europe	2 (14%)
South America	1 (7%)
Africa	1 (7%)
Years of experience (*n* = 14)	Number of participants, *n* (%)
1–5	4 (29%)
6–10	5 (36%)
11–15	2 (14%)
16+	3 (21%)
Mean [range]	9.5 [3.5–17]
Staff size of organisation (*n* = 14)	Number of participants, *n* (%)
Less than 100	4 (29%)
101–250	2 (14%)
251–500	2 (14%)
501–1000	1 (7%)
More than 1000	5 (36%)
Primary affiliation organisation (*n* = 16)^a^	Frequency of responses, *n* (%)
Non‐profit organisation	9 (56%)
Governmental organisation or agency	3 (19%)
Professional association	3 (19%)
Academic institution	1 (6%)
Type of role in guideline programme (*n* = 18)^a^	Frequency of responses, *n* (%)
Guideline methodologist^b^	6 (33%)
Director	6 (33%)
Systematic review/research team^c^	2 (11%)
Subject matter expert	1 (6%)
Other	3 (17%)
Member size of organisation (*n* = 13)^a^	Frequency of responses, *n* (%)
Not a member organisation	4 (31%)
Less than 1000	2 (15%)
1000–5000	2 (15%)
5001–15,000	1 (8%)
15,000–30,000	1 (8%)
More than 30,000	3 (23%)
Type of collaboration engaged in (*n* = 28)^a^	Frequency of responses, *n* (%)
Networking^d^	3 (11%)
Cooperation^e^	6 (21%)
Coordination^f^	7 (25%)
Coalition^g^	5 (18%)
Full collaboration	7 (25%)

^a^ More than one response per participant was recorded for this variable.

^b^ Guideline methodologist: Expert in guideline development methods and processes, including systematic reviews and translation of evidence to recommendations.

^c^ Systematic review/research team: Responsible for conducting systematic reviews and other evidence synthesis products to support the development of a guideline.

^d^ Networking: No formal agreement; informal sharing of information for the mutual benefit of the organisations but decisions are made independently.

^e^ Cooperation: No formal agreement; organisations support each other's activities but are not engaged in a mutual project and decisions are made independently.

^f^ Coordination: Formalised links but each organisation retains autonomy; organisations are engaged in mutual project(s) with some shared decision making.

^g^ Coalition: Formalised links but each organisation retains autonomy; organisations are engaged in mutual project(s) and all members have a vote in decision making.

### 3.1 Overview of Themes

The thematic analysis of interview transcripts identified interconnected barrier, facilitator, and cross‐cutting themes and sub‐themes. Barrier themes captured sub‐themes and codes linked to hindered or halted collaboration, while facilitator themes captured those linked to enhanced collaboration. Cross‐cutting themes captured sub‐themes and codes that aligned as either facilitators or barriers depending on contexts and perspectives described by different interviewees. Themes and sub‐themes are described below, and presented in a themes map (Figure 1). Representative interview quotes, that served as codes for themes and sub‐themes are detailed in Tables 2a, 2b, 2c.

**Table 2a:** Facilitator themes and sub‐themes with representative interview quotes.

3.2 Facilitator themes	
**3.2.1 Templates and technologies**	
3.3.1a Templates (e.g., Terms of Reference, Memorandum of Understanding, protocols, contract templates, conflict of interest information collection templates etc.)	*“Again, that was a learning experience for us. I think at the end of the day, from a process perspective, what we realised is we should write this process down, so that, if, in the future, we were to do this, we need to follow the process all the way because there were some issues that came up with ʻthis is how we approach it,ʼ and ʻthis is how we approach it.ʼ There's a fair bit of back and forth” [Interview 16]*
	*“a template MOU that—if we can avoid the back‐and‐forth with the lawyers, that really helps, so we've developed a template that applies to either an organisation taking the lead or an organisation participating; If we can agree on the common template—which we developed one” [Interview 1]*
	*“We developed a form where there was standard language ‐ and ask them to disclose whatever conflict they had, so that's something we had to do up front” [Interview 11]*
3.3.1b Technologies (e.g., Telecom technologies such as Zoom and MS team, Platforms that allow for virtual collaboration (e.g., Rayyan, Distiller, COVIDENCE, Box, Google Docs, RevMan, GRADEpro, MAGICapp, Sharepoint); Multiuser virtual workspaces, Project and literature/evidence management software)	*“It solidified a move to virtual and Zoom meetings and more effective virtual and Zoom meetings. That was a good thing, I think, that everyone thought they could not accomplish before. I think we've established now that we can get a lot more accomplished via Zoom than anyone ever thought.” [Interview 3]*
	*“Collaboration—I think project management software tools can be helpful. This is something we're trying to get into the space of ‘cause guideline development is a lot about project management and figuring out which parties involved where and who can do what. I think more resources around that; The other element is using some type of project management software. I think that would have helped us a lot because there's so many moving parts, and managing all that is very tough” [Interview 11]*
	*“Regarding software, we've got access to typical software, but that's kind of linked to Cochrane. It's linked to RevMan Web. It's linked to Covidence software, SR Distiller. That kind of software, reference management software, the inherent things that is part of the gears of turning a guideline over. Survey software if you wanna do surveys and so forth, and data………important software like GRADEpro and CINeMA, NMA level software, we also have access to that, and of course telecommunication software” [Interview 4]*
**3.2.2 Initial scoping and planning of the collaboration**	
3.3.2a Type of collaboration engaged (e.g., Networking, Cooperation, Coordination, Coalition, Full collaboration (lead vs. joint))	“*defined joint efforts as one group commits to taking a lead, and one or more groups commit to participation….There's roles and responsibilities for each type of organisation……We do different models (of collaboration); lead‐versus‐participating setup works really well; You really need one coordinating body. That was a pivotal experience that led us to this participating versus lead organisation;” [Interview 1]*
3.3.2b Clarity on “collaboration why, what, when, where, with whom” (e.g., purpose/goals, type and level of collaboration e.g., engagement/participation/division of work), roles and responsibilities, resource allocation, COI disclosure, timelines, logistics, coordination	*“It's really around making sure the goals align, and then would need to figure out, within our workflow, what the bandwidth is of folks within my team to actually work on that collaboration; I would want clear expectations on what the output of their work is, what the questions are. I would wanna know what the timeframe of the work is, and what the mutual expectations would be. What is my team supposed to be doing? What is their team supposed to be doing?” [Interview 8]*
	*“Yes, we can strategise in a group meeting at the beginning of the process. We agree with the method that will be used” [Interview 13]*
	*“if you're gonna collaborate on a topic or on some aspect of a guideline with another group, is just the logistics around know what type of agreements do you need in place. Do you need data‐sharing agreements? Do you need agreements around intellectual property? Sometimes, maybe even just thinking—trying to think through the process.” [Interview 14]*
	*“When you are onboarding these partners, telling them that this is how we develop guidelines, this is usually what the process is, and this is where you're gonna come in, and this is what your role is gonna be in this big picture” [Interview 11]*
**3.2.3 Organisational guideline topic prioritisation**	
	*“[organisation] developed a formal mechanism to determine what topics we will engage in joint efforts with us as the lead or with us as the participating organisation. formal topic prioritisation that we conduct on an annual basis; if somebody wants to do a joint effort with us, if it's on our prioritised list, we say, ʻWe're happy to take the lead. It's on our list. Come along, and we'll do it as a joint effort.ʼ If it's not on our list, we say, ʻWe can't lead anything, but we're happy to follow and be a participating organisation.ʼ That really helps us determine our resource allocation;” [Interview 1]*
**3.2.4 Culture of collaboration**	
3.2.4a Desire/willingness to collaborate	*“My own personal feeling is if we're going to call this a collaborative guideline, let's actually be collaborative and try to be as receptive as possible to allowing input because that's how we get new ideas and fresh perspectives, and why else would we be inviting another group in” [Interview 3]*
3.2.4b People	*“culture beats vision any day. Right? If you have healthy culture within the collaborative group and strong vision leadership, then I think that that truly helps……culture within organisations need to be acknowledged, because you might have a great vision and plan and timetable, but if the people you're working with is a struggle to work with, then it just—you don't wanna do it again, and you'd rather not; I would say that some institutional cultures are just so different that collaborating becomes a heartache, for lack of a better word” [Interview 4]*
**3.2.5 Recognising collaboration benefits**	
3.2.5a Multidisciplinary teams (e.g., diversity of disciplines, expertise, experience, considerations, and perspectives) Note: multidisciplinary teams subtheme captures diversity in experience and expertise, whereas sharing of resources subtheme captures the sharing HR resources and tasks)	*“We have four full‐time methodologists that are on board. They've all got educational backgrounds or master's in public health most of them……I've got a staff member who worked at a local hospital, a large hospital for a couple of years and that's where most of her background is before she came to XXX to work at the more national level guideline development. The other developers have between three and seven years of work at another medical specialty society, so developing guidelines with grade at that same national perspective” [Interview 7]*
	*“We strive to be multidisciplinary, so, if the topics are of interest to professionals in other medical fields, we include those professionals on the workgroup as well. We require at least one patient representative or advocate to sit on our panel to lend their expertise, We do employ outside contractors who have specific expertise in evidence synthesis. These are generally on the evidence‐based practice center or another for‐profit organisation that specialises in health care evidence synthesis.” [Interview 10]*
	*“We're a mix of project management, operations, and then we have more of the technical staff, so the methodologists, …help with the systematic reviews, reviewing or doing the evidence appraisal, helping with developing the draft guidelines or the draft sections” [Interview 2]*
	*“We have a group of a variety of different staff. It consists of guideline methodologists…informatician because that person will help us translate guidelines, at least clinical practice guidelines, into practice…fellows who are gonna help us evaluate guidelines…Implementation staff or other groups, SMEs [Subject Matter Experts]” [Interview 11]*
	*“The broad makeup is technical/medical and project management.” [Interview 15]*
3.2.5b Expand guideline dissemination (e.g., dissemination networks and platforms)	“*Ability to reach more stakeholders and make a bigger impact………if we have collaborated with another professional organisation, we would also be able to really have a broader reach and broader impact in terms of making a positive contribution that gets used” [Interview 5]*
	*“I think the level of collaboration is gonna impact the dissemination. For the one guideline where I mentioned we had co‐sponsors, that was heavily—we had the partner's name, the logo—there's a lot, so the dissemination for that was not just through XXX channels, but through their channels too” [Interview 11]*
	*“if they'll be unaware. They'll become aware eventually, but it does give that extra stamp of endorsement. I think we do reach certain groups of people that we may not have reached through our channels. When you see something coming through your medical professional society saying, “Hey, here's this new document,” versus just XXX putting it out and you may or may not see that, I think there's a difference. It helps. I think that there's benefit. It does have more reach.; What we have found is then, collaborators or liaison organiations will take that advice and really actively promote it or endorse it post hoc. I suppose the more integrated your collaboration is, the more likely you are to see that kind of—or to seek joint dissemination and promotion of the guidance implementation”. [Interview 12]*
3.2.5c Resource sharing (e.g., staff, human resources, access to technology, protocols, templates, budget) Note: multidisciplinary teams subtheme captures diversity in experience and expertise, whereas sharing of resources subtheme captures the sharing human resources and tasks, budgets, technologies/products)	*“We came to find out that in another group, they were really good at translating guidelines using informatics. In our particular division, we had no resources in this area, so we formed a partnership with them.” [Interview 11]*
	*“I would like to see the focus on collaboration be more at the evidence synthesis level. I feel like there's a lot more flexibility in the ability to co‐support or work on collaboratively the evidence synthesis portion to support guidelines.” [Interview 10]*
	*“I mean the university itself has all the university resources, and that includes access to world‐leading library resources, for example. Right? Access to all the databases that one would want access to do evidence synthesis or to find information that you need to find.” [Interview 4]*
	*“I think another big benefit would be sharing resources. This time of budget cuts, many places and groups, a lot of cuts to budgets and things. This would just be great for being able to share resources. That is one more tangential benefit” [Interview 5]*
	*“Regarding the amount of the work, I think that this inter‐collaborative work may reduce the workload for individuals and societies….the human resources to do the work needed for guideline development” [Interview 6]*
3.2.5d Improved guideline comprehensiveness/quantity	*“Particularly if it's your own professional association involved, then I think there's likely to be greater acceptance or greater thought that it's—it was developed by people who know what this profession is like.” [Interview 5]*
	*“If we develop three rapid guidelines per year……the other society, a second society will develop also three guidelines per year. Well, if the model is going to be successful then yes, we're going to produce more guidelines….will have developed three guidelines within a year, and will have participated in another six or seven or eight guideline projects, and the same counts for the other societies as well” [Interview 6]*
	*“Recognising that is a really good way for us to expand the number of guidelines recommendations in our portfolio without drawing heavily on our resources.” [Interview 7]*

**Table 2b:** Barrier themes and sub‐themes with representative interview quotes.

3.3 Barrier themes	
**3.3.1 Misalignment of guideline development purpose, scope, and goals**	
3.3.1a Different expectations for the collaboration	*“In a couple of cases, we went back to our review and approval body, and they said, ‘Why are we doing this? I'm not interested in this.’ One was a pediatric guideline. They said, ‘Our core membership is adult. We're not the experts to comment on review and approval of a pediatric guideline.’ They rejected that. They said, ‘We're not interested’ ” [Interview 1]*
	*“biggest barrier is really just different expectations. Everybody's got slightly different ways of working. It's natural for us to come to the conclusion that our way of working is the best way……Yeah. Those misaligned expectations I feel like can become a barrier” [Interview 7]*
	*“We also turned down a collaboration with another group who is developing guidelines in the same area based on the perspective…They were approaching the topic from an allergy perspective. We, of course, want to approach the topic from a dermatology perspective” [Interview 10]*
3.3.1b Different guideline focus and considered perspectives (e.g., population(s) of interest, target audiences)	*“One of the things, other challenges that I've seen for collaborating, and it revolves around how we define the scope…. Where I see that as a potential, presenting problems is when we go to talk with another specialty society about collaborating, you run into these senses of ownership. Where this is our topic, this is our subject, we're the experts here there's no clear definition of what's the scope of our portfolio, how are we going to manage it, where do we wanna say things?… I see conflict happening every, 'cause then you have to sort out those ownership issues, those intellectual ownership issues, every time you wanna have a collaboration.”* *[Interview 7]*
**3.3.2 Misalignment of guideline development process and methodology**	
3.3.2a Conflicts of interest consideration (unclear (or undisclosed) and management	*“I think in terms of barriers … is do your methods [COI] align? One thing in particular that's really important…is patient representatives on the guideline panels, but not all other organisations agree with that…. There's certain things that we have to do because of our own government and our bylaws and such, and other groups don't.” [Interview 5]* *“ I think it can also be trickier with collaborations'cause you have to agree upon a lot of methodology pieces versus just kind of plowing forward on your own… and there are no the same way to manage our [The the conflict of interest].” [Interview 5]* *“I've seen a lot of projects thwarted by one organisation having more rigorous standards for participation in terms of industry relationships and financial and intellectual conflicts of interest than the other organisation. This is especially difficult if you're trying to collaborate internationally” [Interview 10]*
3.3.2b Evidence appraisals, how evidence is considered and weighted	*“That was a barrier to collaboration. The fact that we were not using GRADE or more modern risk of bias tools, and evidence appraisal was very tangibly a barrier to international collaboration…… I think that using a common base methodology is really helpful. It does facilitate that.” [Interview 5]*
	*“That was problematic, and we had to actually do another subsequent review to capture those more qualitative studies… we had our guideline development group members do the critical appraisal on those references. I guess it's hard when we work with others…. how they do their systematic reviews” [Interview 9]*
3.3.2c Guideline review and approval	*“others aren't as equipped to be as nimble. It might be twice a year that their board meets, and their board is the review and approval body. ‘Are you telling me we have to wait six months until the next review and approval?’ That's a hard thing to get past.” [Interview 1]*
**3.3.3 Guideline collaborations takes greater time or effort**	
	*“One of our challenges at the very beginning was we used to say yes to everybody. ‘Hey, you wanna do a joint—’ ‘Sure. I guess so.’; At the time when you had those four organisations, 25 percent each, and it took 7 years, apart from—I don't let joint efforts become too much of our portfolio just 'cause they're more time and effort. No matter what, they're gonna add on at least two months to development time and probably more” [Interview 1]*
	*“when you get so many people in the room, in addition to internal staff, decision making can be very tricky because what one organisation feels very strongly about, another one may not or may actually feel otherwise, and so negotiating those conversations was very—it took a lot of skill to figure out what the right thing to do was” [Interview 11]*
	*“The traditional models of collaboration, I think, take more time. There is a lengthy contracting period that usually adds one to three months at the onset of the project; There's additional administrative tasks that need to happen to collaborate with members outside of your organisation and coordinate with the other organisation's guideline manager and the like; I think it adds a bit of time to the process.” [Interview 10]* *“Usually, collaborative guidelines developed by X take much longer compared to those that are being developed by X alone” [Interview 6]*
	*“There's two other organisations we had attempted to collaborate with. One, we actually made some headway at first, but it took a—it was a long time trying to get the MOU agreed upon. We kind of got to the point where it was about to get signed, but the other group pulled out.” [Interview 5]*

**Table 2c:** Cross‐cutting themes and sub‐themes with representative interview quotes.

3.4 Cross‐cutting themes	
**3.4.1 Past experiences**	
3.4.1a Positive experiences, facilitating collaboration	*“We have our common partners in crime. Most of the folks on staff came out of the X shop, the X Guideline Development shop. We collaborate quite often back and forth with them just on a one‐to‐one basis—ʻOh, we'll take this one. You take that one.ʼ ʻWe'll take this. You take thatʼ—just to divvy up the workload.”* *[Interview 1]* *“For example, X we've been in collaboration with them for years and years and years 'cause our teams gel well together”* *[Interview 4]*
3.4.1b Negative experiences, inhibiting collaboration	*“I always keep a mental tally of "How did the last joint effort work out with this group? Was it a hassle, or was it a pleasant experience?"* *[Interview 1]* *“Part of the reason they've done it [GL development] on their own is because of a somewhat bad taste that these other collaborations have left in terms of complications”* *[Interview 3]*
**3.4.2 Guideline publication location (e.g., journal publication, professional websites)**	
3.4.2a Publication location, facilitating collaboration	*“it's them coming to us, seeing if we'll collaborate with them, 'cause we have a pretty wide exposure through the Journal X and the X organisation name.; That's why people wanna join up with us, too, 'cause they can access some of those things that we do for all of our guidelines….XXX for example, doesn't care about publishing in their own journal. They don't have a journal. Instead of searching around for a journal, they're happy to publish in ours;* *[Interview 1]* *When we are collaborating and we're the lead, it's gonna be published in our journal, it'll follow really our normal route; When it's an externally led collaboration we're very much hands‐off on the journal submission aspect. They'll handle all of that*. *[Interview 7]*
3.4.2b Publication location, inhibiting collaboration	*“If we talk about the collaborative projects, then it is a substantial barrier that we—it is difficult to manage a simultaneous publication in different journals because other societies have different official journals. They have different affiliated journals, …. This is a potential barrier because the different journals are handled by different publishing offices, by different publishers. We cannot operationalise a simultaneous publication for the benefit of members… I suspect, this may be a barrier in collaboration between societies for the purposes of guideline development.”* *[Interview 6]* *“It's often tricky to figure out a publication strategy 'cause journals, of course, want unique content. It's not simply a matter of publishing”* *[Interview 10]*
**3.4.3 Levels of knowledge and training**	
3.4.3a Levels of knowledge and training, facilitating collaboration	*“That process worked out well because they also followed GRADE in the guideline development process to a significant degree. In areas where they were not following GRADE, they were willing to learn more about GRADE, allow GRADE to be incorporated in the guideline development process”* *[Interview 16]* *“I think training is always helpful. Particularly, and we have actually done a little bit of this in our team too to help us wrap our heads around some of these things, if you are looking at adopting or adapting someone else's guideline….Sometimes, it is more helpful if you can get direct training from experts with your guideline panel to understand these processes.”* *[Interview 14]*
3.4.3b Levels of knowledge and training, inhibiting collaboration	*It would be probably a barrier to collaboration with another organisation if they're at a point that I needed to suggest training. I would be hoping and expecting that the folks there are up to speed on grading methods and such”* *[Interview 8]*

### 3.2 Facilitator Themes

Several themes that facilitate collaboration in guideline development were identified and included: use of templates and technologies; initial scoping and planning; organisational guideline topic prioritisation; culture of collaboration; and recognition of the benefits of collaboration.

#### 3.2.1 Templates and Technologies

This theme organised templates and technologies that were mentioned to enhance collaboration into two distinct sub‐themes. The templates sub‐theme captured various literature, such as protocols and manuals, that outline guideline development processes and methodology and offered templates that allowed for their standardised application. Templates on memoranda of understanding (MOU) and agreement (MOA), sample letters of agreement, terms of reference documents, contract templates, COI disclosure/assessment templates were specifically mentioned by interviewees. The use of pre‐approved templates with standard language was described to streamline collaborations by reducing the ʻback and forthʼ and promoting consistency and concordance among collaborators.

The technologies sub‐theme captures virtual workspaces, telecommunication technologies, project management and systematic review management software, which interviewees indicated to have played a role in enhancing their collaborative guideline development work. Technologies such as MS Teams, Zoom, Box, Google Docs, Sharepoint, Rayyan, DistillerSR, COVIDENCE, RevMan, GRADEpro Guideline Development Tool, and MAGICapp were reported to have been helpful during collaborative guideline development.

#### 3.2.2 Initial Scoping and Planning of the Collaboration

Subthemes captured within the theme of initial planning and scoping included discussions on the type of collaboration engaged (e.g., Networking, Cooperation, Coordination, Coalition, Full collaboration) and clarity on collaboration process (e.g., roles, responsibilities, resource allocation, COI disclosures, timelines, outputs). Most interviewees found initial scoping and planning of the proposed guideline development work supports collaboration, particularly through the consideration of elements such as where, when, why, how, and with whom to collaborate. Scoping and planning activities were explained to clarify and support consensus building on critical aspects of a guideline development process and avoid misalignments (Appendix S1).

#### 3.2.3 Organisational Guideline Topic Prioritisation

Prioritisation of topics for guideline development and work planning at the organisational level emerged as a separate but closely connected extension of the initial scoping and planning facilitator theme. Interviewees noted internal planning and prioritisation improved decision making at the organisational level to pursue collaborations more efficiently. This included more carefully considering which collaborations to pursue based on internal priorities and how to allocate organisational resources. Internal planning and prioritisation was also found to enhance organisational support and approval of guidelines with external collaborators.

#### 3.2.4 Culture of Collaboration

A culture of collaboration was an overarching theme present in the majority of interviewee recounts of positive collaboration experiences. This theme captured the desire to collaborate with organisations that are receptive to and actively support internal and external collaborations. Characteristics such as flexibility, open‐mindedness, strong vision and leadership were often mentioned when discussing positive collaborative cultures.

#### 3.2.5 Recognising Collaboration Benefits

Interviewees often mentioned various benefits of collaborative guidelines to be facilitators for collaboration. Collaboration benefits were organised into distinct sub‐themes, described below.

The establishment of multidisciplinary teams was a major benefit of collaborative guideline development. Multidisciplinary teams formed during collaborations were noted to bring together individuals from diverse disciplines with a range of expertise, experiences, considerations and perspectives. Individuals described past experiences where clinicians, methodologists, project managers and administrators worked synergistically to develop guidelines.

The bringing together of experts from relevant areas was also described to increase a guideline's reach among more audiences and acceptance by more guideline users. Collaboration was thought to help expand guideline dissemination leading to increased use of a guideline's content. This was an often identified benefit of collaboration.

Sharing of resources was another benefits sub‐theme. This captured the sharing of staff and workloads from a human resources perspective, as well as the sharing of funding, budgets, guideline development resources, tasks, and evidence review outputs. An example mentioned by a guideline developer of a national government organisation was sharing of evidence review outputs, e.g., screened reference lists, systematic review findings, and evidence summaries with another organisation involved in similar guidance development. In this example, evidence review outputs were shared to inform the development of two guidelines tailored to two different regions.

Another benefit sub‐theme was improvements to guideline comprehensiveness and quantity. Interviewees explained collaboration allowed for improved guideline quality because of combined expertise, perspectives, contributions, and efforts. Interviewees mentioned collaboration reduced duplication, enabling the production of a greater number of guidelines and more efficient use of resources.

### 3.3 Barrier Themes

Three main themes emerged from the analysis as barriers to guideline development collaboration: misalignment of purpose, scope, goals; misalignment of guideline development process and methodology; and the perception that collaboration takes greater time and effort.

#### 3.3.1 Misalignment of Guideline Development Purpose, Scope, and Goals

Interviewees provided examples that illustrated how collaborative guideline development could become infeasible or ineffective as a consequence of ʻunclear expectations and scopeʼ about the collaboration, or lack of clarity on a collaborative guideline's ʻownershipʼ (refer to Appendix S1). Differences in a guideline's focus and considered perspectives, stemming from different target populations (e.g., paediatric vs. adult patient populations) and/or target audiences (e.g., allergists vs. dermatologists), were captured within this theme.

#### 3.3.2 Misalignment of Guideline Development Process and Methodology

Guideline developers spoke about occasions where collaboration was stifled due to guideline development process or methods being different or incongruent. This theme captured subthemes linked to COI, certainty or weighting of evidence, guideline review and approval. Two examples of misalignment provided by interviewees included differences in collaborating organisations' rigour in gathering and assessing COI leading to ʻ*thwartedʼ* collaborations; and lack of agreement on risk of bias methods used.

#### 3.3.3 Guideline Collaborations Takes Greater Time or Effort

Multiple interviewees mentioned a belief that collaboration takes more ʻtime and effortʼ, with the initiation of collaborative projects requiring discussion, agreement, and approvals among more stakeholders and participants. This was perceived to lead to extended timelines and possible increases in coordination and administrative efforts. One interviewee stated that their organisation limits the number of joint efforts on guideline activities with other organisations because in their past experiences, such collaborations took more time and effort.

### 3.4 Cross‐Cutting Themes

Several cross‐cutting themes emerged from the analysis, which contained sub‐themes that could be considered collaboration facilitators or barriers depending on interviewee provided context and examples.

#### 3.4.1 Past Experiences in Collaborative Guideline Development

Positive experiences were reported to facilitate additional collaborations due to familiarity between collaborators or understanding of involved organisational processes. However, past negative experiences in collaboration had the opposite effect, and limited or prevented future collaborative guideline development.

#### 3.4.2 Guideline Publication Location

The publication journal or website of the completed work had both positive and negative effects on guideline collaboration. Some interviewees mentioned a greater desire to collaborate with organisations that had established journals or dissemination avenues in which to share the completed guidelines. In contrast, different approval and peer‐review requirements by different journals of collaborating organisations, and inability to duplicate a guideline publication in different journals were noted barriers.

#### 3.4.3 Levels of Knowledge and Training

The degree of knowledge and training on guideline development process and methodology among collaborators was also captured in facilitator and barrier sub‐themes. This cross‐cutting theme was tied to the barrier theme on misalignment of guideline development processes and methodology, and the facilitator theme on initial scoping and planning of collaboration. Knowledge gaps in guideline development best practice processes and methodologies, such as approaches to evidence reviews, evidence appraisals and analysis methods, were noted to pose barriers to successful collaboration. Other interviewees noted that similar understanding of guideline development methodologies and processes among guideline development participants enabled collaborations to operate smoothly. Based on these experiences, many interviewees advocated for guideline development training and education (e.g., GRADE training) for collaborators at the initial stages to ensure similar knowledge levels and reduce misalignments.

## 4 Discussion

### 4.1 Summary of Findings

Barriers and facilitators to collaboration are interconnected, and successful collaborations are dependent on alignment in process, methodology and expectations, along with the sharing of resources between guideline developers and organisations. These findings reflect known best practices in guideline development, such as priority setting, the use of standard processes and the establishment of agreed upon rules of engagement before starting to work together.

### 4.2 Strengths and Limitations

To the knowledge of the study authors, this is the first study to qualitatively explore collaborative guideline development through interviews among seasoned experts with confirmed experience and expertise in this area. As such, it is anticipated the inductive approach to the thematic analysis supported the generation of themes and sub‐themes reflective of interviewee experiences and insights, while the GCWG research team's knowledge and interest in this area enriched the analysis and interpretation of interview data.

The generalisability of findings could be limited because most interviewees (85%) reported working within guideline development organisations in North America and Europe, which likely lead to overrepresentation of experiences and insights from these regions.

### 4.3 Principal Findings

The analysis builds on previous findings to explore themes linked to collaboration among an international sample of guideline developers using qualitative interview data [16, 20]. Consistent with the needs assessment findings and other research on this area, this analysis found additional resources, time and efforts related to collaborative guideline development were significant barriers, whether perceived or real [16]. This study revealed that there was willingness to engage in collaborative guideline development when pro‐collaborative cultures were present among guideline developers and their organisations, and collaboration benefits were realised. Benefits like the efficient use of resources, balanced consideration of perspectives and evidence, harmonised processes and recommendations, and improved guidance dissemination previously described as benefits of collaboration [11, 12, 13, 16, 20] are further confirmed by this study.

The value of multidisciplinary team compositions are well established in guideline development literature [1, 2, 3, 4, 5, 6, 7, 8, 9]. A systematic review comparing group compositions in healthcare consensus development found representation from relevant disciplines and stakeholders allowed for the consideration of ranging perspectives, reduced bias, and enhanced decision making reliability and quality [20]. Another systematic review of literature from a variety of disciplines found committee performance on decision making regarding highly technical issues was improved when groups took diverse opinions into account [21]. This practice also enhanced credibility, widespread acceptance, and implementation of committee decisions [21].

Past collaborative experiences had the potential to both positively and negatively influence collaboration in guideline development [22]. The influence of existing guideline developer connections on further collaboration was a notable study finding, echoed by another recent study on living guideline development [23]. Cheyne and colleagues reported collaborations in living guidelines often built on pre‐existing connections or evolved from prior collaborations [23].

Multiple interviewees also discussed how early agreement on scope and approach to guideline development, before embarking on the guideline development work, were extremely beneficial for collaboration. Specifically, discussion and decision on levels of collaboration, guideline development cycle processes and timelines, through formal agreements were mentioned facilitators. As such, guideline developers embarking on collaborative work involving multiple guideline development organisations are strongly encouraged to reference, agree upon and implement formal agreements and process [1, 2, 3, 4, 5, 6, 7, 8, 9, 10, 11, 12, 13]. The GCWG has developed a toolkit of resources to help plan and negotiate collaborations, and address identified needs of guideline developers and barriers associated with collaboration (available from https://macgrade.mcmaster.ca/resources/gin-guideline-collaborations-toolkit-hub/) [24]. The toolkit is informed by previous research conducted by the GCWG involving guideline developers [12, 16, 25].

Levels of knowledge and training among collaboration participants was a cross‐cutting theme identified, meaning similar levels of knowledge regarding guideline development cycle processes facilitated collaboration while inconsistent knowledge impeded collaboration. Additionally, the leverage of technologies and templates were mentioned to facilitate efficient collaboration. The International Guideline Training & Certification Program (INGUIDE), Grading of Recommendations, Assessment, Development, and Evaluations (GRADE), Cochrane Evidence Essentials training modules, and Appraisal of Guidelines for Research and Evaluation (AGREE) along with various project and evidence management software offer trainings to support guideline development methods and best practice [26, 27]. The adoption of collaborator training as on‐boarding and continuous learning activities could mitigate barriers linked to misalignments.

## 5 Conclusions

This study identified facilitators and barriers of collaborative guideline development, many of which appear to be interconnected. Early discussion and agreement on collaboration scope and processes was an important facilitator, while misalignments in a collaboration's scope, goals, and guideline development cycle processes were the greatest barriers. Pro‐collaborative cultures among guideline developers and organisations, recognising benefits such as enhanced guideline quality, quantity, utilisation, expertise and resource sharing, foster collaboration. Positive experiences, similar knowledge and understanding of guideline development facilitated collaboration, while negative experiences and inconsistent knowledge were barriers. These findings collectively highlight the need to first negotiate and plan processes and intended methodologies, establish collaborative agreements, and provide training supports for participants when developing guidelines. Future research should investigate quantifying the relative importance of themes and sub‐themes among different guideline developers and organisations, and elucidating the interconnections between the identified barriers, facilitators and cross‐cutting themes.

## Author Contributions

Conceptualisation: Toju Ogunremi, Rebecca L. Morgan, Murad Alam, Pamela Ginex, Christina Ventura, Robyn Temple‐Smolkin and Priya Jakhmola. Development of interview questionnaires: Toju Ogunremi, Rebecca L. Morgan, Amanda Graham, Murad Alam, Pamela Ginex, Christina Ventura, Robyn Temple‐Smolkin and Yasser Amer. Interviewers: Toju Ogunremi, Rebecca L. Morgan, Chatura Prematunge, Amanda Graham, Murad Alam, Michael Yi, Umer Nadir, Robyn Temple‐Smolkin, Christina Ventura, Pamela Ginex, Priya Jakhmola, Emily Senerth, Emma McFarlane and Heba Hussein. Data coding and analysis: Chatura Prematunge, Aggie Bak, Amanda Graham and Bright Huo (Toju Ogunremi was involved in confirmation of final sub‐themes and themes). Writing (original draft): Chatura Prematunge, Amanda Graham, Toju Ogunremi, Aggie Bak and Bright Huo. Writing (review and editing): All authors.

## Conflicts of Interest

Rebecca L. Morgan, Yasser Sami Amer and Holger Schunemann are members of the Editorial Board of Clinical and Public Health Guidelines but did not participate in the review or the editorial process of this article, and had no influence on the Editorial decisions related to it.

## Supporting information

Appendix Pre Interview Quesionnaire and Interview Guide.

## Data Availability

Data underlying the study are not publicly available due to data containing potentially identifying information. Participants provided informed consent to use their questionnaire responses and interview data for the purposes of this study project. Participants did not agree to sharing of this data publicly. Therefore, this data is not publicly available.
