# Supplementary material for: Facilitators and Barriers to Successful Collaboration in Guideline Development: Experiential Insights of Collaborative Guideline Developers
Source: Clin Public Health Guidel. 2025 Aug 26;2(4):e70036. doi: 10.1002/gin2.70036 (PMC13563340; doi:10.1002/gin2.70036)
Supplement: Supplementary file 1 [file GIN2-2-e70036-s001.docx]

**GIN Guidelines Collaboration Working Group:**

Pre-Interview Survey

Introduction

Collaboration among organizations and groups that produce clinical practice guidelines is important to move recommendations to the point of care. Despite the benefits of collaboration, it does not occur routinely. To better understand the complexities, benefits, and barriers to collaboration an interprofessional group of Guidelines International Network (GIN) members are conducting interviews with guideline developers. **The primary goal of this project is to learn and share best practices related to guideline collaboration to improve best practices among GIN members.** The interview should take approximately 30 minutes.

The findings from this project will be developed into an internal toolkit on guideline collaboration resources for use by GIN members, with the aim of improving collaboration processes. The project findings may also be presented at future GIN conferences and inform peer-reviewed publications submitted for publication and presentation at the GIN Conference. To minimize privacy concerns, all results will be de-identified. No names or organizations will be linked to a specific response but if you wish, your organization can be listed as participating.

Demographic Questions

What type of organization do you work for?

☐Non-profit

☐Professional Association

☐Governmental

☐Academic

☐For Profit

☐Other (please describe)

What is your role in your guideline development program?

☐Guideline methodologist

☐Subject matter expert

☐Other panel member

☐Systematic review/research team

☐Secretariat for guideline development groups (admin arm)

☐Director

☐Other: Click or tap here to enter text.

How long have you been at your organization?

Click or tap here to enter text.

In your current position, have you collaborated on guideline activities with other organizations?

☐Yes

☐No

**Collaboration may include:*

*- Two or more organizations or parts of organizations working together to either develop an entire set of guidelines or some component of a larger guideline such as a specific topic review, specific recommendations, defining the scope of the project, etc.*

**-***Jointly adapting existing guidelines for novel uses or updating existing guidelines.*

**Note:** *Collaboration does not need to be exactly equivalent or symmetrical in the role of the parties and one organization could have a greater leadership role.*

If no, why?

Click or tap here to enter text.

If yes, which best describes the type of collaboration you engaged in with other organizations?

| ☐ | Networking: No formal agreement; informal sharing of information for the mutual benefit of the organizations but decisions are made independently |
| --- | --- |
| ☐ | Cooperation: No formal agreement; organizations support each other’s activities but are not engaged in a mutual project and decisions are made independently |
| ☐ | Coordination: Formalized links but each organization retains autonomy; organizations are engaged in mutual project(s) with some shared decision making |
| ☐ | Coalition: Formalized links but each organization retains autonomy; organizations are engaged in mutual project(s) and all members have a vote in decision making |
| ☐ | Full collaboration: Formalized links, which are written in a signed agreement (MOU); organizations work towards consensus on decision making to achieve a shared vision |
| ☐ | Don’t know |
| ☐ | Other (please specify): Click or tap here to enter text. |

What region is your organization headquartered in?

☐African

☐North America

☐South America

☐European

☐Eastern Mediterranean/Middle Eastern

☐Southeast Asia

☐Western Pacific

What is the staff size of your organization? (Total number of staff)

☐Less than 100

☐101 – 250

☐251-500

☐501-1000

☐More than 1,000

What is the member size of your organization?

☐Less than 1,000

☐1,000-5,000

☐5,001 – 15,000

☐15,001 – 30,000

☐More than 30,000

☐Not a member organization (governmental or other)

How many guideline development groups are within your total organization?

Click or tap here to enter text.

On average, how many staff are on each guideline group?

Click or tap here to enter text.

On average, how many volunteers (members, patients, others) are on each guideline?

Click or tap here to enter text.

collaboration amongst guideline developers

commonly implemented approaches and barriers encountered

gin Guidelines collaboration working group

Table of Contents

[Survey Introduction	2](#_Toc79519190)

[Instructions for Survey Administrator	2](#_Toc79519191)

[Guideline Collaboration Questions	3](#_Toc79519192)

Survey Introduction

Thank you very much for agreeing to participate in the Guideline International Network Collaboration Working Group’s project, titled, “Collaboration Amongst Guidelines Developers: Commonly Implemented Approaches and Barriers Encountered.” **The primary goal of this project is to learn and share best practices related to guideline collaboration to improve best practices among GIN members.** We would like to better understand guideline collaborations and the factors that may lead to success or failure.

The following survey contains a series of questions related to your experience with collaboration in guideline development as well as the general guideline development process. These questions should take approximately 30 minutes of your time.

Participant responses will be recorded and transcribed and the findings from this project will be developed into an internal toolkit on guideline collaboration resources for GIN members. The project findings may also be presented at future GIN conferences and inform peer-reviewed publications. . Your responses are voluntary and should be based on your experiences. Your name and organization will not be linked to your specific responses, but if you wish, your organization can be listed as participating.

Instructions for Survey Administrator

On the next page, please find the list of interview questions in bold. Question prompts are provided below each bolded question in italics. Only after the participant has had adequate time to respond to the bolded question should the prompts be given.

Guideline Collaboration Questions

| 1. Tell us about the  (a) type of staff your guideline development groups employ,  (b) their expertise, and  (c) how these groups are funded. | [Answer] |
| --- | --- |
| 2a. What resources are available to support your work in guideline development?  2b. Are there specific resources available to support collaboration?  2c. What resources would you recommend to people who work in guideline development? Are there any specific trainings/resources you would recommend to improve collaboration? | [Answer] |
| 3a. In the intro and demo questionnaire, you indicated ‘yes’ to the question “In your current position have you collaborated on guideline activities with other organizations?”; is there a recent (last 3 years) or current collaboration you can speak to? | Yes  No |
| Note to Interviewer: Skip to question 11 if respondent answers “No.” | |
| 3b. Can you tell us more about that collaboration?   - *When was this? (Past or current/recent project)* - *How was this level of collaboration decided?* - *What were the benefits and disadvantages of your level of collaboration?* - *What strategies facilitated collaboration?* - *What were the challenges of collaboration?*   4. Considering all guidelines you have collaborated on, what barriers have you experienced when collaborating?   - *Disagreements about scope, interpretation of evidence, composition of expert panels or Disclosures of Interest (DOI), personality conflicts, disagreements about financial or other resource investment, lack of agreement about timeline, poor communication, lack of expertise, lack of either content or methodology expertise in some of the participating groups, etc.* - *If you have avoided collaboration, what are the reasons (i.e., adequacy of internal resources, prior unsuccessful collaborations, impracticality or expense of collaborations)?* - *What would you or will you do differently in the future?* | [Answer] |
| 5. Can you provide any specific examples or case studies of challenges to collaboration that you have experienced that you haven’t previously discussed?   - *This includes examples that resulted in either a successful or unsuccessful collaboration* |  |
| 6. When collaborating, how do you assess and manage Conflict of Interest (COI) of the Guideline Development Group members from different organizations?   - *What tool or document do you rely on to capture COIs?* - *What types of conflicts does this assess (e.g., financial, academic, intellectual, institutional, etc.)?* - *What is the process for declaring a COI?* - *What is the process when a member declares a COI (e.g., excluded from the group, limited discussions, rescued from specific topics)?* - *Are disclosures reassessed?*   - *If so, how often?*   - *For each project?* | [Answer] |
| 7. When collaborating, how is external/peer review conducted?   - *Is the process different than when not collaborating?* - *Are external reviews completed by organizations and/or individuals?* - *What is the selection process for identifying external reviewers?* - *How much time is allocated for external review?* - *Are the guidelines formally endorsed and/or affirmed by external organizations?* - *Is your review for a) clinical content, b) methodology, c) both, d) other?* | [Answer] |
| 8. When collaborating, how do you manage publication, dissemination, and implementation of the guidelines?   - *Is there a difference in the implementation process of a guideline document when collaborating with organizations than when not collaborating?*   - *Difference in procedure?*   - *Difference in challenges – time needed for publishing, approval process, etc.?* - *Do you think that collaboration help to reach groups which would otherwise be unaware of the guidelines being published?* | [Answer] |
| 9. When collaborating, how do you manage updating/retiring of guidelines?   - *Is the process different than when not collaborating?* | [Answer] |
| 10. What was your experience when collaborating with other organizations during the COVID-19 pandemic?   - *Were resources increased/decreased during this time?* - *Did you collaborate on any guidelines specific to COVID-19?* - *Did you need to adapt methodology/processes and if so, how was this agreed upon?* - *Was your collaboration different than usual?* - *Were any other guidelines affected as a result and how was this managed with collaborators?* - *What were your barriers/facilitators to collaboration during the COVID-19 pandemic?* - *Did the COVID-19 pandemic identify any areas for improvement in your collaborative guideline development process?* | [Answer] |
| 11. How do you see yourself approaching collaboration in the future?   - *Do you think you would be collaborating in the future? If yes, do you want to collaborate more often? At which level?* - *Are there any additional reasons you want to collaborate more, aside from the benefits of collaboration previously mentioned?*   - *Speedier time to completion?*   - *Resource use?*   - *Producing a superior product?*   - *Greater acceptance of the final product?* | [Answer] |
| 12. Would you like to add anything else? | [Answer] |

---------- END OF INTERVIEW ----------
